# Anisotropic Hyperfine Interaction of Surface-Adsorbed Single Atoms

**DOI:** 10.1021/acs.nanolett.2c02782

**Published:** 2022-11-01

**Authors:** Jinkyung Kim, Kyungju Noh, Yi Chen, Fabio Donati, Andreas J. Heinrich, Christoph Wolf, Yujeong Bae

**Affiliations:** † Center for Quantum Nanoscience (QNS), Institute for Basic Science (IBS), Seoul 03760, South Korea; ‡ Department of Physics, Ewha Womans University, Seoul 03760, South Korea; § Ewha Womans University, Seoul 03760, Republic of Korea

**Keywords:** Scanning tunneling microscopy, Electron spin resonance, Scanning probe, Hyperfine interaction, Nuclear spin

## Abstract

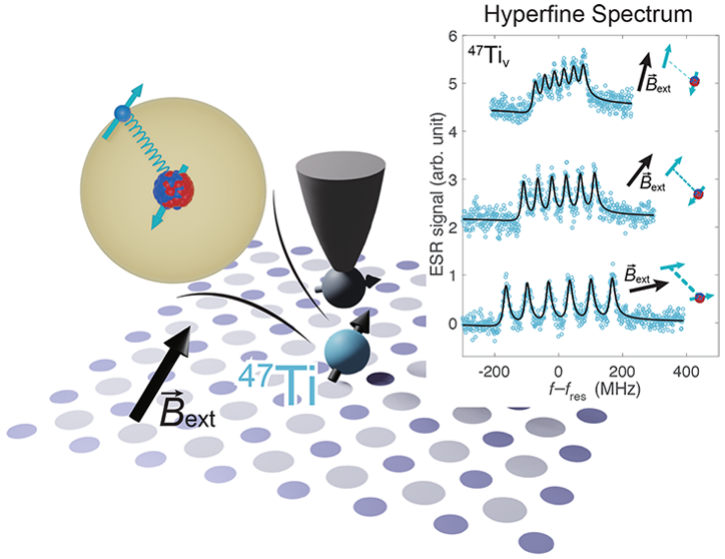

Hyperfine interactions have been widely used in material science, organic chemistry, and structural biology as a sensitive probe to local chemical environments. However, traditional ensemble measurements of hyperfine interactions average over a macroscopic number of spins with different geometrical locations and nuclear isotopes. Here, we use a scanning tunneling microscope (STM) combined with electron spin resonance (ESR) to measure hyperfine spectra of hydrogenated-Ti on MgO/Ag(100) at low-symmetry binding sites and thereby determine the isotropic and anisotropic hyperfine interactions at the single-atom level. Combining vector-field ESR spectroscopy with STM-based atom manipulation, we characterize the full hyperfine tensors of ^47^Ti and ^49^Ti and identify significant spatial anisotropy of the hyperfine interactions for both isotopes. Density functional theory calculations reveal that the large hyperfine anisotropy arises from highly anisotropic distributions of the ground-state electron spin density. Our work highlights the power of ESR-STM-enabled single-atom hyperfine spectroscopy in revealing electronic ground states and atomic-scale chemical environments.

Conventional ensemble magnetic resonance techniques have been extensively used for probing hyperfine interactions between paramagnetic spin centers and nearby nuclear spins, where the sensitivity largely depends on the spin concentration.^1^ Hyperfine interactions at the single spin level have recently attracted significant interest due to promise in sensitive detection of the local chemical environment^2^ and nuclear-spin-based quantum information processing.^3−6^ These new scientific endeavors are enabled by technological developments that allow electron spin resonance (ESR) operations at the single-spin level,^7^ for example, through optically addressable color centers in insulators^8,9^ or semiconductor donor atoms equipped with nanofabricated charge detectors.^10^ However, for a general paramagnetic center placed in its native chemical environment, characterization of the hyperfine interactions at the single-spin level has been extremely difficult.

Scanning tunneling microscopy (STM) with ESR capabilities offers a new appealing platform for *in situ* characterization of individual spin-carrying atoms and molecules.^11−13^ ESR-STM spectroscopy offers tens of nanoelectrovolt energy resolution, far beyond traditional STM bias spectroscopy,^14−16^ thus allowing for probing hyperfine interactions at the atomic scale.^17,18^ When combined with STM’s single-atom selectivity, hyperfine interactions from single atoms with different isotopes and different binding sites can be individually determined without spatial averaging.^19^

Here we use a state-of-the-art ESR-STM system to measure the full hyperfine tensor of single hydrogenated ^47^Ti and ^49^Ti atoms on MgO/Ag(100). Using a vector magnetic field and STM-based atom manipulation, we quantify the isotropic and anisotropic hyperfine interactions for the two Ti isotopes. A large hyperfine anisotropy of more than 67% is observed for both Ti isotopes on a low-symmetry binding site, which indicates a highly anisotropic distribution of the ground-state spin density that is consistent with density functional theory (DFT) results.

We performed ESR-STM experiments using a home-built STM system equipped with radio-frequency (RF) cabling and a two-axis vector magnet.^20^ The vector magnet provides an in-plane magnetic field up to 9 T and an out-of-plane field up to 2 T. Individual Ti atoms were deposited on two monolayers of MgO(100) grown on a Ag(100) substrate while the sample was kept below 10 K in the STM stage (Figure 1a). Evaporation of Ti was performed using a commercial electron-beam evaporator. Ti rods with natural isotope abundance were used in which the most abundant isotope, ^48^Ti, has zero nuclear spin, while ^47^Ti (with 7.4% abundance) has a nonzero nuclear spin of *I* = 5/2 and ^49^Ti (with 5.4% abundance) has a nuclear spin of *I* = 7/2 (Figure 1b).^21^ Due to the residual hydrogen gas in the vacuum chamber,^22^ Ti atoms deposited on the MgO surface are most likely hydrogenated and have an electron spin-1/2.^23,24^ Hereafter, we denote the hydrogenated Ti atoms as Ti for simplicity. Ti atoms were found at two different binding sites on MgO(100), atop of an oxygen atom^23,25^ or at a bridge site between two oxygen atoms.^24,26^ In this work, we focus on Ti atoms on the bridge binding sites, which have a significantly larger hyperfine coupling than the oxygen binding sites.^17^ Note that in our experimental setup, the in-plane magnetic-field axis is 15.5° tilted from the oxygen lattice direction of MgO(100) (Figure 1a). This results in two nonidentical bridge binding sites of Ti, referred to as Ti near the vertical direction (Ti_v_) and Ti near the horizontal direction (Ti_h_).^20,27^ These two inequivalent in-plane sites, in combination with the two-axis magnetic field, allow us to determine the hyperfine interaction tensor as discussed below.

**Figure 1:**
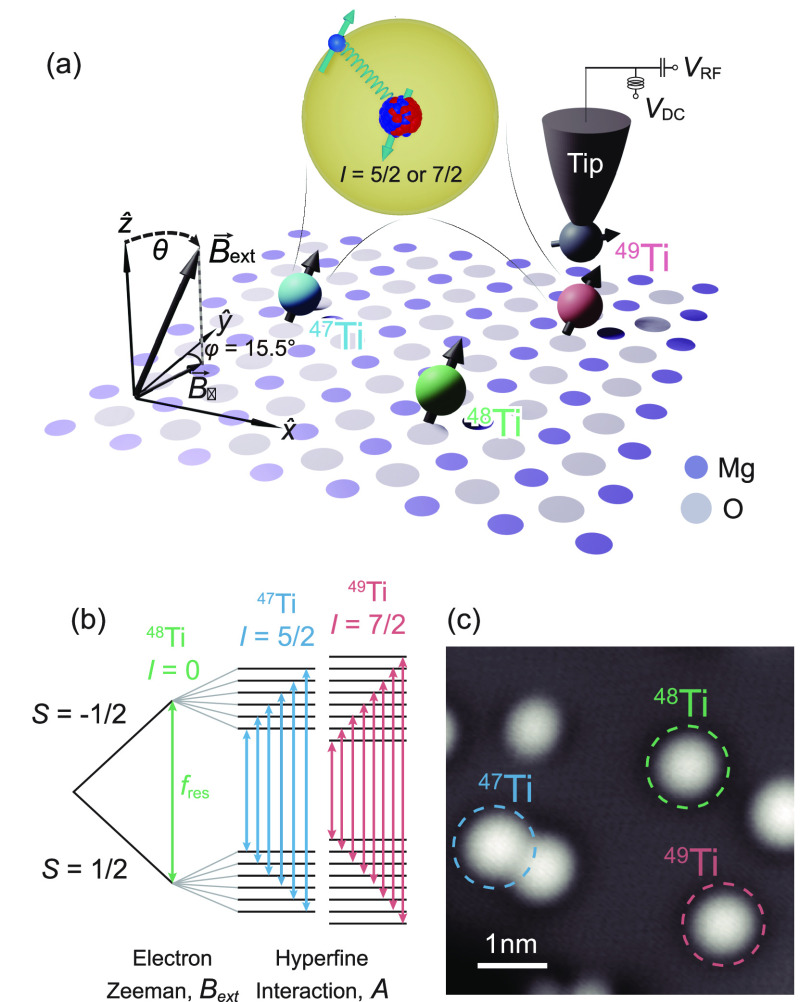
Hyperfine interaction of single hydrogenated titanium (Ti) atoms on the MgO/Ag(100) surface. (a) Schematic of the ESR-STM measurements on different isotopes of Ti under a rotatable magnetic field. Single-atom ESR spectroscopy is performed by detecting the change of spin-polarized tunnel current under resonant driving. The MgO lattice directions are indicated by *x̂* and *ŷ*, whereas *ẑ* is the out-of-plane direction. The vector external magnetic field ***B***_ext_ is applied in a plane which is 15.5° rotated from the *yz*-plane around the *ẑ*-axis. The rotation angle of ***B***_ext_ relative to the out-of-plane direction is labeled as θ. (b) Schematic of the energy levels and ESR transitions of different Ti isotopes in the presence of a magnetic field *B*_ext_ and the hyperfine interaction *A*. The ESR transitions are denoted by double sided arrows. (c) STM image showing three different types of Ti isotopes. Different Ti isotopes show identical topographic features but can be readily identified through ESR spectroscopy (Figure 2 and S1) (*V*_DC_ = 100 mV, *I* = 20 pA).

Figure 1c shows a typical STM image containing three different types of Ti isotopes. While Ti atoms with different isotopes show identical STM topographic and bias spectroscopic features, they are clearly distinguishable from STM-based ESR spectroscopy (Figure S1). Out of 94 Ti atoms that we measured, the majority (83 atoms) show only one ESR peak, corresponding to the most abundant ^48^Ti isotope with zero nuclear spin (Figures 1b and S1). Six Ti atoms (roughly 6.4%) exhibit 6 ESR peaks that correspond to ^47^Ti with *I* = 5/2, and 5 Ti atoms (roughly 5.3%) exhibit 8 ESR peaks that correspond to ^49^Ti with *I* = 7/2 (Figures 1b and S1). The hyperfine splitting is much smaller than the thermal energy at 0.6 K, resulting in nearly equal populations in the nuclear spin states and thus equal peak intensities. The ESR peaks observed in our measurements are found to be equally spaced (see, e.g., Figures 2 and S1), which indicates negligible contributions from the nuclear Zeeman interaction and the electric quadrupole interaction.^28^ These considerations allow us to write down a simplified spin Hamiltonian of the ^47^Ti and ^49^Ti atoms as
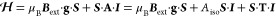
1where ***S*** and ***I*** are the electron and nuclear spin operators, respectively, ***B***_ext_ is the external magnetic field, and **g** is the electron *g*-tensor. **A** is the tensor of the hyperfine interaction that can be decomposed into an isotropic contact term, *A*_iso_, that originates from the direct overlap between the electronic wave function and the nuclear spin, and an anisotropic contribution, **T**, that originates from the dipolar interaction between the electron and nuclear spins.^28^ The principal axes for **g** and **A** tensors coincide with the crystalline lattice axes of MgO due to the *C*_2*v*_ symmetry of Ti on the bridge site.^28^

**Figure 2:**
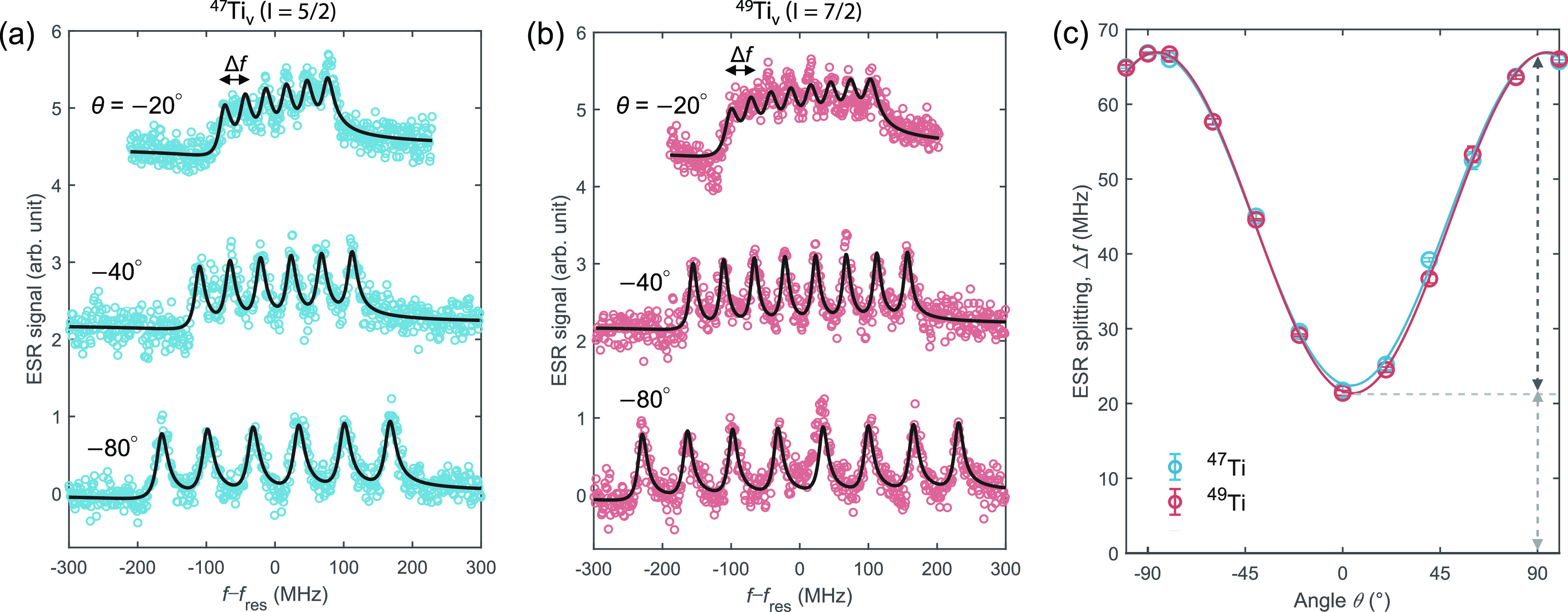
Angular variations of the hyperfine interactions of ^47^Ti_v_ and ^49^Ti_v_. (a) Hyperfine spectra of ^47^Ti_v_ and (b) ^49^Ti_v_ measured at different angles θ of the external magnetic field. For both isotopes, the hyperfine splitting is greatly increased when the field is rotated away from the out-of-plane direction (i.e., for the shown data, toward more negative θ). Fits in (a) yield a hyperfine splitting of 30.0 ± 0.4 MHz at θ = −20°, 44.4 ± 0.3 MHz at θ = −40°, and 66.5 ± 0.2 MHz at θ = −80°, from a 95% confidence interval of the fit to this data set. In (b), the hyperfine splitting is determined to be 29.0 ± 0.3 MHz at θ = −20°, 44.6 ± 0.1 MHz at θ = −40°, and 65.8 ± 0.2 MHz at θ = −80°. ESR spectra are plotted against *f* – *f*_res_, where *f*_res_ is the resonance frequency of a ^48^Ti_v_ atom (with zero nuclear spin) measured under the same conditions. ESR spectra are normalized in intensity, and successive curves are vertically shifted for clarity (*V*_DC_ = 40 mV, *I*_set_ = 1.5–8 pA, *V*_RF_ = 15–50 mV). (c) The hyperfine splittings of ^47^Ti_v_ and ^49^Ti_v_ measured as a function of the magnetic field angle, θ. Solid curves are guides to the eye. Dashed arrows highlight the significant hyperfine anisotropy between in-plane and out-of-plane directions. Error bars are given by the standard errors of different measurements under the same conditions. The statistical error from different tip/atom combinations is ∼0.8 MHz (Figure S3b). From these measurements we conclude that there is no noticeable difference in the hyperfine splittings of ^47^Ti and ^49^Ti.

Different components of the **A** tensor can be probed by changing the direction of the external magnetic field ***B***_ext_. Under the external magnetic field ***B***_ext_ of 0.8 T (which is used in all measurements shown in the main text), the electron Zeeman term of the Hamiltonian in eq 1 dominates and determines the electron spin direction to be almost parallel with ***B***_ext_ (some deviation arises from **g**-factor anisotropy^20^). The electron spin direction in turn determines the nuclear spin direction through the hyperfine coupling **A**. The rotation of ***B***_ext_ thus collectively rotates the electron spin ***S*** and the nuclear spin ***I***, allowing us to probe different components of **A** through the term ***S***·**A**·***I***. The energy change associated with the hyperfine interaction, ***S***·**A**·***I***, is detected through the splitting between adjacent ESR peaks, *Δf*. For a Ti atom with *S* = 1/2, the ESR transition associated with a certain nuclear spin state, *m*_*I*_, has a frequency of *hf*_*m*_*I*__ = μ_B_*B*_ext_*g*(θ) + *A*(θ) *m*_*I*_, where *h* is the Planck’s constant and *g*(θ) and *A*(θ) are the experimentally probed *g*-factor and hyperfine constant, respectively, at the field angle θ. The frequency splitting between adjacent ESR lines thus directly yields the hyperfine interaction, since *Δf* = |*f*_*m*_*I*__ – *f*_*m*_*I*_ ± 1_ | = *A*(θ). The relation between the experimentally probed *A*(θ) and the principal values of the **A** tensor will be discussed later (see eq 2).

Figure 2 shows the experimental results at different field angles. At θ = −20° (close to the out-of-plane direction, see Figure 1a), the ESR splittings for ^47^Ti_v_ (Figure 2a) and ^49^Ti_v_ (Figure 2b) are measured to be 30.0 ± 0.4 MHz and 29.0 ± 0.3 MHz, respectively, where the error bars are determined from a 95% confidence interval of a fit to this data set. Analyses of different tip/atom combinations yield a statistical uncertainty of ∼0.8 MHz (Figure S3b). We find that the energy splittings *Δf* measured for ^47^Ti_v_ and ^49^Ti_v_ are equal within the uncertainty of our measurements, as expected from their identical electronic ground states and very similar nuclear magnetic moments.^29^ The hyperfine interaction is significantly increased when we rotate the magnetic field closer to an in-plane direction as shown in Figure 2a,b. The large angular variations of the hyperfine splittings for ^47^Ti_v_ and ^49^Ti_v_ are summarized in Figure 2c. In both cases, the largest splitting of ∼67 MHz is observed at θ ≈ 90° when ***B***_ext_ is applied along an in-plane direction, while the smallest splitting of ∼22 MHz is obtained along the out-of-plane direction. These observations indicate very large hyperfine anisotropy (around 67%, see Figure 2c) for both ^47^Ti_v_ and ^49^Ti_v_ atoms. Due to their nearly identical hyperfine interactions, we will focus on ^47^Ti in the following.

To determine the hyperfine interaction along the third axis that is not in the tunable plane of the external magnetic field (Figure 1a), we exploit two inequivalent bridge binding sites of Ti on MgO (Ti_v_ and Ti_h_). Using atom manipulation, Ti atoms can be moved reversibly between different binding sites on MgO.^23^Figure 3a,b shows STM images of ^47^Ti_v_ and ^47^Ti_h_ taken before and after atom manipulation. Strikingly, the binding site significantly changes hyperfine spectra as shown in Figure 3c,d. With ***B***_ext_ applied along the out-of-plane direction (at θ = 0°), Ti_v_ and Ti_h_ are at physically identical sites and exhibit similar hyperfine splittings (upper curves in Figure 3c,d). With ***B***_ext_ applied along the in-plane direction, however, the large anisotropy observed in Ti_v_ is almost absent in Ti_h_ (lower curves in Figure 3c,d). This trend is clearly shown in Figure 3f where a large change of the hyperfine splitting is only observed for Ti_v_. Since the in-plane field direction of Ti_v_ is almost aligned with the O–Ti–O direction (referred to as the O–O direction hereafter, see the inset of Figure 3c), this trend suggests that the hyperfine interaction along the O–O direction is significantly larger than that along the out-of-plane and Mg–Ti–Mg directions (the latter referred to as the Mg–Mg direction hereafter).

**Figure 3:**
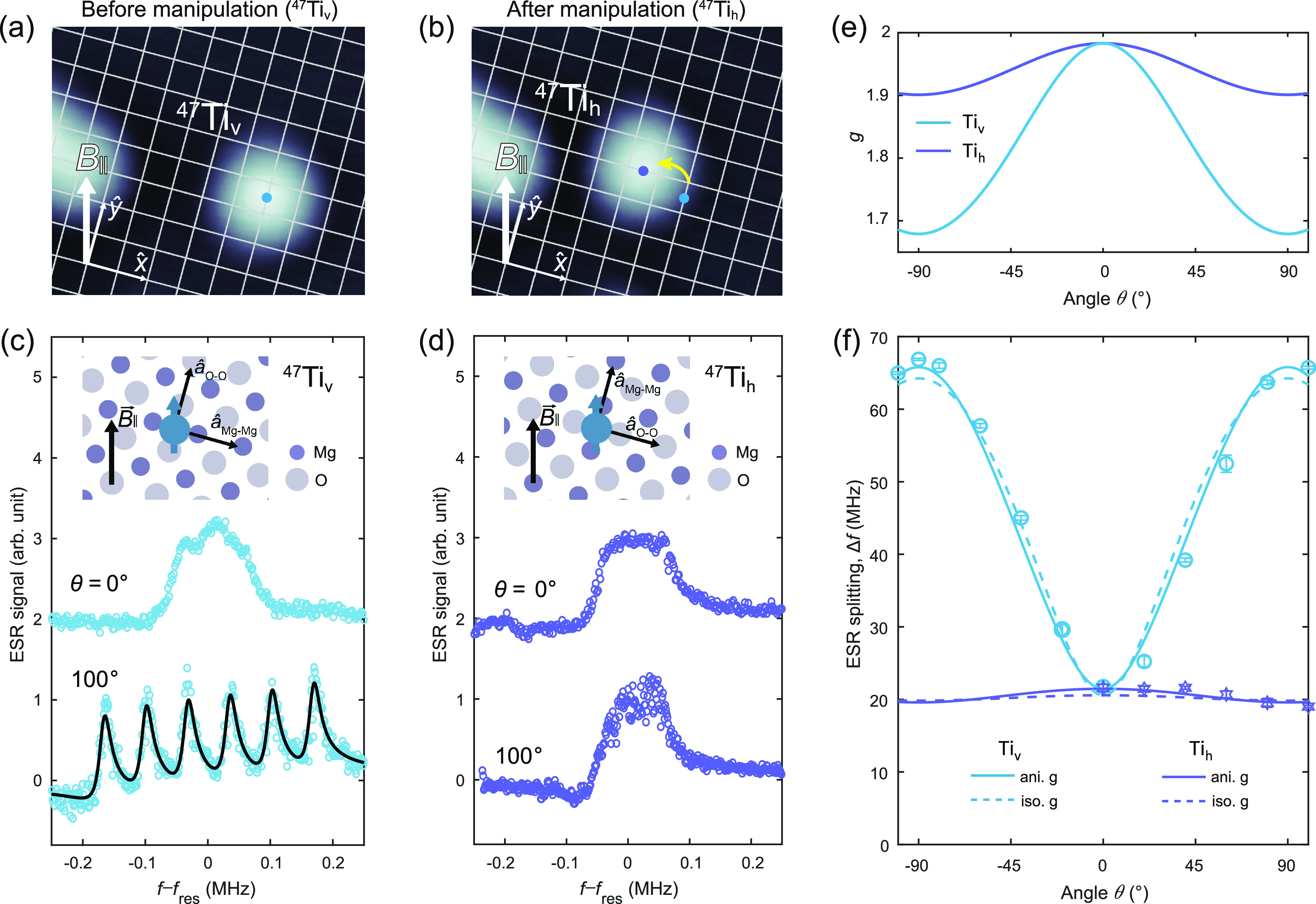
Binding-site-dependent hyperfine spectra of Ti atoms on MgO. (a) STM image of ^47^Ti at a vertical bridge binding site (^47^Ti_v_). The intersections of grid lines correspond to the locations of oxygen atoms in the MgO lattice. (b) STM image of ^47^Ti_h_ taken after moving ^47^Ti_v_ in (a) by 1.5 × 0.5 oxygen lattices, to the horizontal site. The thick white arrow in each STM image indicates the in-plane magnetic field direction (***B***_∥_) with respect to the MgO lattice (scan conditions: *V*_DC_ = 100 mV, *I* = 20 pA). (c) Hyperfine spectra of ^47^Ti_v_ and (d) ^47^Ti_h_, measured at θ = 0° (upper) and 100° (lower). The curves are normalized to unity. Successive curves are vertically shifted for clarity (*V*_DC_ = 40 mV, *I* = 12–20 pA, *V*_RF_ = 15–20 mV). Insets show that ***B***_∥_ is close to the O–O direction (i.e., the O–Ti–O direction) for ^47^Ti_v_ and Mg–Mg direction (i.e., the Mg–Ti–Mg direction) for ^47^Ti_h_. (e) *g*-factors of the bridge-site Ti as a function of the angle θ as calculated based on the anisotropic *g*-factors determined in ref (20). (f) Anisotropic hyperfine coupling of ^47^Ti_v_ and ^47^Ti_h_ measured at different magnetic field angles. The O–O direction, close to the in-plane direction of ^47^Ti_v_, shows a significantly larger hyperfine interaction strength than the other two principal axes. Solid and dashed curves correspond to fits to eq 2 with anisotropic and isotropic g-values, respectively. The hyperfine splittings at small field angles in Figure 3f are obtained using a different STM tip that allows better resolution (see Figure S4).

The extensive hyperfine spectra presented above allow us to quantitatively extract the hyperfine interactions along the three principal axes of bridge-site Ti atoms. A and g components along the O–O direction are denoted as A_O_ and g_O_, respectively (similarly for the Mg–Mg direction). Taking both **A** and **g**-factor anisotropies under consideration, the experimentally observed hyperfine splitting *A*(θ) is related to its principal values by^28^
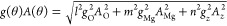
2where (*l*, *m*, *n*) are the direction cosines given by (*l*, *m*, *n*) = (sin θ cos φ, sin θ sin φ, cos θ) and *g*(θ) = (*l*^2^*g*_O_^2^ + *m*^2^*g*_Mg_^2^ + *n*^2^*g*_*z*_^2^)^1/2^. In our setup, φ_h_ = 15.5° and φ_v_ = 105.5° for ^47^Ti_v_ and ^47^Ti_h_, respectively. Using the *g*-factors that we measured before on bridge-site Ti, (*g*_O_, *g*_Mg_, *g*_*z*_) = (1.653, 1.917, 1.989) (Figure 3e),^20^ the best fits to the hyperfine splittings (solid curves in Figure 3f) yield three principal values for the hyperfine interaction tensor, (*A*_O_, *A*_Mg_, *A*_*z*_) = (68.97 ± 1.23, 11.66 ± 3.59, 21.23 ± 1.52) . These results indicate a significantly larger hyperfine interaction along the O–O axis compared to the other two axes (Mg–Mg and out-of-plane directions), agreeing with the trends in Figure 3f. The *g*-factor anisotropy is not important in determining the hyperfine constants, as shown by very similar fitting results assuming an isotropic *g*-factor of 2.003 (dashed curves in Figure 3f). The complete determination of the hyperfine tensor **A** allows us to calculate its isotropic and anisotropic components (see eq 1). Since the dipolar hyperfine tensor **T** is traceless, the isotropic hyperfine interaction can be determined experimentally to be *A*_iso_ = 1/3Tr(**A**) = 33.95 ± 1.36 MHz. The dipolar hyperfine tensor **T** is then obtained by subtracting *A*_iso_ from **A**, resulting in (*T*_O_, *T*_Mg_, *T*_*z*_) = (35.02 ± 1.84, – 22.29 ± 3.84, – 12.72 ± 2.04) MHz.

The observed large hyperfine anisotropy along the O–O direction reflects a highly anisotropic ground-state wave function of bridge-site Ti atoms. This is confirmed by DFT calculations using the GIPAW formalism implemented in Quantum Espresso.^30,31^ Our model consists of 4 layers of Ag capped by two layers of MgO and a hydrogenated Ti atom adsorbed on a bridge site (Figures 4a and S8). The resulting DFT ground-state is a mixture of *s*, *d*_*yz*_, and *d*_*x*^2^–*y*^2^_ orbitals, and its isosurface of spin-polarization is shown in Figure 4a (details about the Ti orbital composition can be found in Figure S9). We found that the isotropic hyperfine interaction, *A*_iso_, is positive and dominates over the dipolar contribution, *T*. As a result, the electron and nuclear spins of Ti are antialigned in the ground state regardless of the magnetic field direction, and the same holds true for their magnetic moments as shown in the upper row of Figure 4b–d (note that the *g*-factors of both the electron and nuclear spins of Ti are negative). The large hyperfine anisotropy along the O–O direction arises from the highly anisotropic ground-state spin distribution (mostly concentrated in the plane spanned by Mg–Mg and *ẑ* directions, see Figure 4a). With the magnetic field applied along the O–O direction, this electron spin distribution results in a positive dipolar term, *T*_O_ > 0, which adds to *A*_iso_ and is responsible for the largest hyperfine interaction along this principal axis (Figure 4b). A magnetic field applied along the Mg–Mg or out-of-plane direction results in a negative dipolar term that reduces the amplitude of the total hyperfine interaction along these directions (Figure 4c,d). Quantitatively, we note that the GIPAW results overestimate the polarization of the 4*s* shell resulting in a Fermi contact contribution that is too large (*A*_iso_ ≈ 170 MHz), but capture the correct trend of the hyperfine anisotropy of the dipolar part (Figure S8). The difference between our results and previously published DFT works for Ti on thin layers of MgO^32,33^ stems from the presence of the Ag substrate as well as the hydrogenated state of Ti. A comparison with refs (32 and 33) highlights the sensitivity of our measurement to the 4*s* and 3*d* state of the adatom. While the DFT result depends on several factors, such as the exchange-correlation functional or static/dynamic corrections to the screening, we find that our calculations well describe the measured anisotropy of the dipolar tensor. The large DFT value of the Fermi contact term, which is indicative of a strong 4*s* polarization, most likely stems from an overestimation of the 4*s*-3*d* hybridization that in turn leads to a repolarization of the 4*s* shell. Importantly, unlike an earlier experimental study^17^ measured with a single-axis magnetic field, the combined use of vector-field ESR spectroscopy and atom manipulation here allows precise characterization of the full hyperfine tensor and hence an accurate determination of the ground-state spin distribution. The experimental measurement of the hyperfine tensor and *g*-factor anisotropy, combined with ab initio calculations, allows for a precise determination of the electronic ground state of a single paramagnetic center.

**Figure 4:**
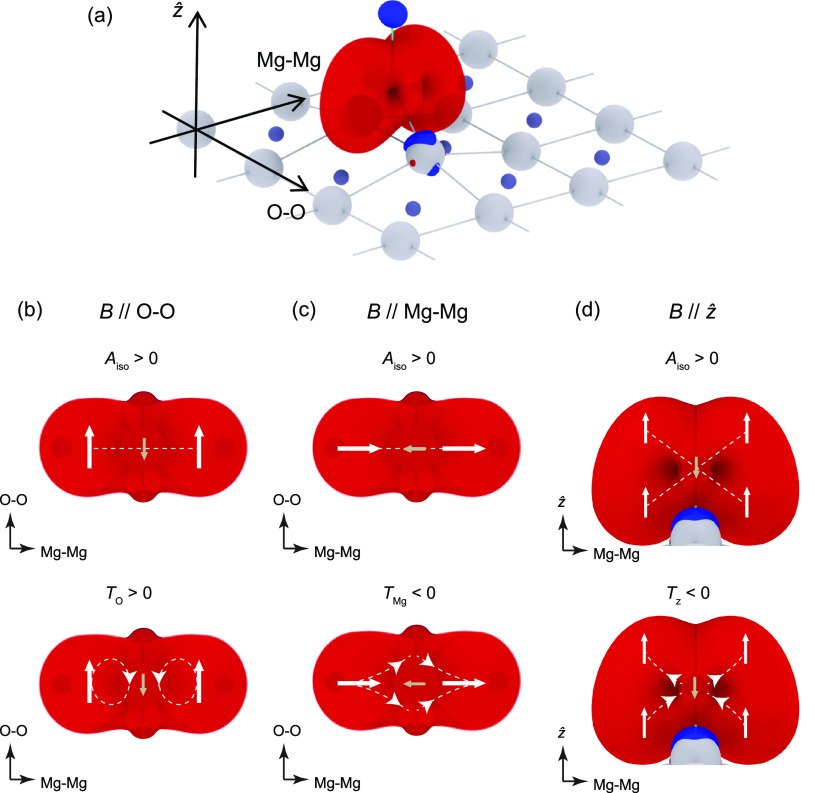
DFT calculations of the spin distribution and hyperfine interactions of bridge-site Ti on MgO. (a) Isometric view of Ti on MgO/Ag(100) with only the top layer of MgO shown for clarity. The red (blue) isosurfaces represent the positive (negative) electron spin polarization (isovalue = 0.002 electrons/*a*_0_^3^). (b–d) Schematics of isotropic and anisotropic hyperfine interactions with ***B***_ext_ applied along the three principal axes. The isotropic hyperfine interaction, *A*_iso_, is positive and dominates over the dipolar contribution, *T*. As a result, the nuclear magnetic moment (brown arrow) is antialigned with the electron magnetic moment (white arrows) in the ground state regardless of the magnetic field direction (upper row in (b–d)) (note that the *g*-factors of both the electron and nuclear spins of Ti are negative). The anisotropic dipolar contribution, on the other hand, modifies the hyperfine strength depending on the direction of ***B***_ext_ (lower row in (b-d)). When ***B***_ext_ is applied along the O–O direction in (b), a positive dipolar term, *T*_O_, adds to *A*_iso_ and is responsible for the largest hyperfine interaction along this principal axis. ***B***_ext_ applied along the Mg–Mg or *ẑ* direction results in a negative dipolar term that reduces the amplitude of the total hyperfine interaction along these directions.

In this work, we show how angle-dependent single-atom ESR-STM spectra enable the precise determination of the hyperfine anisotropy and hence ground-state electron distribution. By taking advantage of the nanoelectronvolt energy resolution, ESR-STM hyperfine spectroscopy adds a unique probe of the local electronic structure to the toolbox of STM. We envision that various types of single spin centers can be probed in a similar fashion.^10^ A local-probe characterization of the nuclear spin bath of quantum dots and donors in semiconductors can provide insight into their decoherence channels. Spins in insulators such as nitrogen-vacancy centers in diamond can in principle be probed in a similar fashion provided suitable metallization. Optimization of the quantum spin properties may facilitate their future applications in spin-based quantum computing and quantum sensing.

During the preparation of this manuscript, we became aware of a similar experiment performed by another group.^34^ The overall experimental data agree well with those presented here, and the extracted hyperfine tensor agrees with our result within the error bars. ref (34). uses a point-charge-based model for their analysis that is complementary to and independent from the DFT simulations presented here, but the two calculations arrive at similar conclusions.

## Supporting Information Available

STM images and ESR spectra of different Ti isotopes on MgO/Ag(100), hyperfine spectra with an equidistant frequency fit, hyperfine splitting as a function of external magnetic field direction measured with different atoms and tips, hyperfine splitting as a function of the magnitude of the external magnetic field, hyperfine splitting as a function of tunnel current, density functional theory and EasySpin calculations of Ti isotopes on MgO/Ag(100) (PDF)

## References

[ref1] Berliner L. J. Spin labeling: theory and applications; Berliner L. J., Ed.; Academic Press: New York, 1976.

[ref2] Lovchinsky I.; Sanchez-Yamagishi J. D.; Urbach E. K.; Choi S.; Fang S.; Andersen T. I.; Watanabe K.; Taniguchi T.; Bylinskii A.; Kaxiras E.; Kim P.; Park H.; Lukin M. D. Magnetic resonance spectroscopy of an atomically thin material using a single-spin qubit. Science 2017, 355, 503–507. 10.1126/science.aal2538. 28104795

[ref3] Kane B. E. A silicon-based nuclear spin quantum computer. Nature 1998, 393, 133–137. 10.1038/30156.

[ref4] Kalra R.; Laucht A.; Hill C. D.; Morello A. Robust Two-Qubit Gates for Donors in Silicon Controlled by Hyperfine Interactions. Phys. Rev. X 2014, 4, 021044 10.1103/PhysRevX.4.021044.

[ref5] Abobeih M. H.; Randall J.; Bradley C. E.; Bartling H. P.; Bakker M. A.; Degen M. J.; Markham M.; Twitchen D. J.; Taminiau T. H. Atomic-scale imaging of a 27-nuclear-spin cluster using a quantum sensor. Nature 2019, 576, 411–415. 10.1038/s41586-019-1834-7. 31853078

[ref6] Zhao N.; Honert J.; Schmid B.; Klas M.; Isoya J.; Markham M.; Twitchen D.; Jelezko F.; Liu R.-B.; Fedder H.; Wrachtrup J. Sensing single remote nuclear spins. Nat. Nanotechnol. 2012, 7, 657–662. 10.1038/nnano.2012.152. 22941402

[ref7] Rugar D.; Budakian R.; Mamin H. J.; Chui B. W. Single spin detection by magnetic resonance force microscopy. Nature 2004, 430, 329–332. 10.1038/nature02658. 15254532

[ref8] Doherty M. W.; Manson N. B.; Delaney P.; Jelezko F.; Wrachtrup J.; Hollenberg L. C. The nitrogen-vacancy colour centre in diamond. Phys. Rep. 2013, 528, 1–45. 10.1016/j.physrep.2013.02.001.

[ref9] Schirhagl R.; Chang K.; Loretz M.; Degen C. L. Nitrogen-Vacancy Centers in Diamond: Nanoscale Sensors for Physics and Biology. Annu. Rev. Phys. Chem. 2014, 65, 83–105. 10.1146/annurev-physchem-040513-103659. 24274702

[ref10] Zwanenburg F. A.; Dzurak A. S.; Morello A.; Simmons M. Y.; Hollenberg L. C. L.; Klimeck G.; Rogge S.; Coppersmith S. N.; Eriksson M. A. Silicon quantum electronics. Rev. Mod. Phys. 2013, 85, 961–1019. 10.1103/RevModPhys.85.961.

[ref11] Baumann S.; Paul W.; Choi T.; Lutz C. P.; Ardavan A.; Heinrich A. J. Electron paramagnetic resonance of individual atoms on a surface. Science 2015, 350, 417–420. 10.1126/science.aac8703. 26494753

[ref12] Zhang X.; Wolf C.; Wang Y.; Aubin H.; Bilgeri T.; Willke P.; Heinrich A. J.; Choi T. Electron spin resonance of single iron phthalocyanine molecules and role of their non-localized spins in magnetic interactions. Nat. Chem. 2022, 14, 59–65. 10.1038/s41557-021-00827-7. 34764471

[ref13] Chen Y.; Bae Y.; Heinrich A. J. Harnessing the Quantum Behavior of Spins on Surfaces. Adv. Mater. 2022, 2107534 10.1002/adma.202107534. 34994026

[ref14] Lambe J.; Jaklevic R. C. Molecular Vibration Spectra by Inelastic Electron Tunneling. Phys. Rev. 1968, 165, 821–832. 10.1103/PhysRev.165.821.

[ref15] Song Y. J.; Otte A. F.; Kuk Y.; Hu Y.; Torrance D. B.; First P. N.; de Heer W. A.; Min H.; Adam S.; Stiles M. D.; MacDonald A. H.; Stroscio J. A. High-resolution tunnelling spectroscopy of a graphene quartet. Nature 2010, 467, 185–189. 10.1038/nature09330. 20829790

[ref16] Delgado F.; Fernández-Rossier J. Inelastic Electron Tunneling Spectroscopy of a Single Nuclear Spin. Phys. Rev. Lett. 2011, 107, 076804 10.1103/PhysRevLett.107.076804. 21902416

[ref17] Willke P.; Bae Y.; Yang K.; Lado J. L.; Ferrón A.; Choi T.; Ardavan A.; Fernández-Rossier J.; Heinrich A. J.; Lutz C. P. Hyperfine interaction of individual atoms on a surface. Science 2018, 362, 336–339. 10.1126/science.aat7047. 30337408

[ref18] Yang K.; Willke P.; Bae Y.; Ferrón A.; Lado J. L.; Ardavan A.; Fernández-Rossier J.; Heinrich A. J.; Lutz C. P. Electrically controlled nuclear polarization of individual atoms. Nat. Nanotechnol. 2018, 13, 1120–1125. 10.1038/s41565-018-0296-7. 30397285

[ref19] Köhler J.; Brouwer A. C. J.; Groenen E. J. J.; Schmidt J. Single Molecule Electron Paramagnetic Resonance Spectroscopy: Hyperfine Splitting Owing to a Single Nucleus. Science 1995, 268, 1457–1460. 10.1126/science.268.5216.1457. 17843664

[ref20] Kim J.; Jang W.-j.; Bui T. H.; Choi D.-J.; Wolf C.; Delgado F.; Chen Y.; Krylov D.; Lee S.; Yoon S.; Lutz C. P.; Heinrich A. J.; Bae Y. Spin resonance amplitude and frequency of a single atom on a surface in a vector magnetic field. Phys. Rev. B 2021, 104, 174408 10.1103/PhysRevB.104.174408.

[ref21] Haynes W. M.; Lide D. R.; Bruno T. J. CRC handbook of chemistry and physics; CRC Press, 2016.

[ref22] Natterer F.; Patthey F.; Brune H. Quantifying residual hydrogen adsorption in low-temperature STMs. Surf. Sci. 2013, 615, 80–87. 10.1016/j.susc.2013.04.008.

[ref23] Yang K.; Bae Y.; Paul W.; Natterer F. D.; Willke P.; Lado J. L.; Ferrón A.; Choi T.; Fernández-Rossier J.; Heinrich A. J.; Lutz C. P. Engineering the Eigenstates of Coupled Spin-1/2 Atoms on a Surface. Phys. Rev. Lett. 2017, 119, 227206 10.1103/PhysRevLett.119.227206. 29286811

[ref24] Bae Y.; Yang K.; Willke P.; Choi T.; Heinrich A. J.; Lutz C. P. Enhanced quantum coherence in exchange coupled spins via singlet-triplet transitions. Science Advances 2018, 4, eaau4159 10.1126/sciadv.aau4159. 30430136 PMC6226279

[ref25] Steinbrecher M.; van Weerdenburg W. M. J.; Walraven E. F.; van Mullekom N. P. E.; Gerritsen J. W.; Natterer F. D.; Badrtdinov D. I.; Rudenko A. N.; Mazurenko V. V.; Katsnelson M. I.; van der Avoird A.; Groenenboom G. C.; Khajetoorians A. A. Quantifying the interplay between fine structure and geometry of an individual molecule on a surface. Phys. Rev. B 2021, 103, 155405 10.1103/PhysRevB.103.155405.

[ref26] Seifert T. S.; Kovarik S.; Juraschek D. M.; Spaldin N. A.; Gambardella P.; Stepanow S. Longitudinal and transverse electron paramagnetic resonance in a scanning tunneling microscope. Science Advances 2020, 6, eabc5511 10.1126/sciadv.abc5511. 32998882 PMC7527223

[ref27] Veldman L. M.; Farinacci L.; Rejali R.; Broekhoven R.; Gobeil J.; Coffey D.; Ternes M.; Otte A. F. Free coherent evolution of a coupled atomic spin system initialized by electron scattering. Science 2021, 372, 964–968. 10.1126/science.abg8223. 34045351

[ref28] Abragam A.; Bleaney B. Electron paramagnetic resonance of transition ions; OUP: Oxford, 2012.

[ref29] Mabbs F. E.; Collison D. Electron paramagnetic resonance of d transition metal compounds; Elsevier: Amsterdam, 1992.

[ref30] Varini N.; Ceresoli D.; Martin-Samos L.; Girotto I.; Cavazzoni C. Enhancement of DFT-calculations at petascale: Nuclear Magnetic Resonance, Hybrid Density Functional Theory and Car-Parrinello calculations. Comput. Phys. Commun. 2013, 184, 1827–1833. 10.1016/j.cpc.2013.03.003.

[ref31] Giannozzi P.; Baseggio O.; Bonfà P.; Brunato D.; Car R.; Carnimeo I.; Cavazzoni C.; de Gironcoli S.; Delugas P.; Ferrari Ruffino F.; Ferretti A.; Marzari N.; Timrov I.; Urru A.; Baroni S. Quantum ESPRESSO toward the exascale. J. Chem. Phys. 2020, 152, 154105 10.1063/5.0005082. 32321275

[ref32] Shehada S.; dos Santos Dias M.; Guimarães F. S. M.; Abusaa M.; Lounis S. Trends in the hyperfine interactions of magnetic adatoms on thin insulating layers. npj Computational Materials 2021, 7, 87 10.1038/s41524-021-00556-y.

[ref33] Tosoni S.; Pacchioni G. Magnetic nature and hyperfine interactions of transition metal atoms adsorbed on ultrathin insulating films: a challenge for DFT. Phys. Chem. Chem. Phys. 2022, 24, 15891–15903. 10.1039/D2CP01224C. 35762384

[ref34] Farinacci L.; Veldman L. M.; Willke P.; Otte S. Experimental determination of a single atom ground state orbital through hyperfine anisotropy. 2022; https://arxiv.org/abs/2207.06037 (accessed 13 Jul 2022). 10.1021/acs.nanolett.2c02783 PMC9650725 36305860

