# Supplementary material for: Anisotropic Hyperfine Interaction of Surface-Adsorbed Single Atoms
Source: Nano Lett. 2022 Nov 1;22(23):9766–72. doi: 10.1021/acs.nanolett.2c02782 (PMC9756343; doi:10.1021/acs.nanolett.2c02782)
Supplement: Supplementary file 1 — nl2c02782_si_001.pdf [file nl2c02782_si_001.pdf]

Supporting Information

Anisotropic hyperfine interaction of surface-adsorbed single atoms

Jinkyung Kim,[1,2,†] Kyungju Noh,[1,2,†] Yi Chen,[1,3,†] Fabio Donati,[1,2] Andreas J. Heinrich,[1,2,*], Christoph Wolf,[1,3,*] and Yujeong Bae[1,2,*]

[1]Center for Quantum Nanoscience (QNS), Institute for Basic Science (IBS), Seoul 03760, South Korea

[2]Department of Physics, Ewha Womans University, Seoul 03760, South Korea

[3]Ewha Womans University, Seoul 03760, Republic of Korea

[†]These authors contributed equally to this work.

*Corresponding authors: A.J.H. (heinrich.andreas@qns.science), C.W. (wolf.christoph@qns.science), Y.B. (bae.yujeong@qns.science)

Table of contents

Section 1: STM images and ESR spectra of different Ti isotopes on MgO/Ag(100)

Section 2: Hyperfine spectra with an equidistant frequency fit

Section 3: Hyperfine splitting as a function of external magnetic field direction measured with different atoms and tips

Section 4: Hyperfine splitting as a function of the magnitude of the external magnetic field

Section 5: Hyperfine splitting as a function of tunnel current

Section 6: Density functional theory and EasySpin calculations of Ti isotopes on MgO/Ag(100)

Section 1. STM images and ESR spectra of different Ti isotopes on MgO/Ag(100)

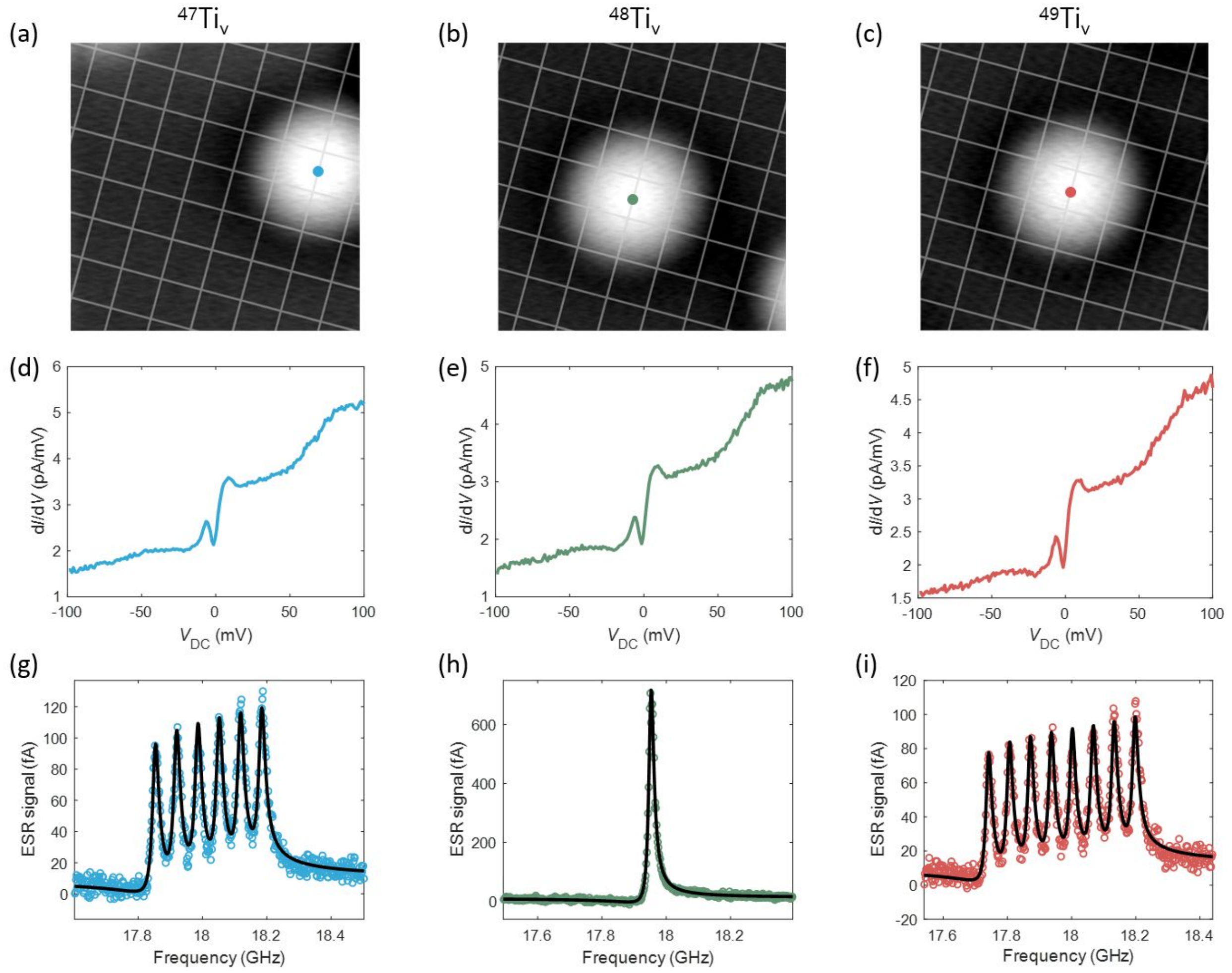

Figure S1. Constant-current STM images ((a), (b), (c)), d*I*/d*V* spectra ((d), (e), (f)), and ESR spectra ((g), (h), (i)) on ^{47}Ti, ^{48}Ti, and ^{49}Ti. STM topographic images and spectra show no noticeable difference between ^{47}Ti, ^{48}Ti and ^{49}Ti. Ti isotopes are clearly distinguishable from ESR spectra: while ^{48}Ti with no nuclear spin shows a single ESR peak, ^{47}Ti and ^{49}Ti show $2I + 1$ peaks due to the hyperfine interaction, where $I = 5/2$ and 7/2, respectively. The solid lines in (g–h) are the same as the hyperfine fits used in the main text. Scan conditions in a–c: $V_{DC} = 100$ mV, $I_{set} = 10$ pA, and scanning size: 2×2 nm^2; d*I*/d*V* measurement

conditions in d–f: V_{DC} = 100 mV, I_{set} = 100 pA, $V_{Lock\text{-}in}$ = 2 mV, and f_{mod} = 333 Hz; ESR measurement conditions in g–i: V_{DC} = 40 mV, I_{set} = 12 pA, and V_{RF} = 15 mV.

Section 2. Hyperfine spectra with an equidistant frequency fit

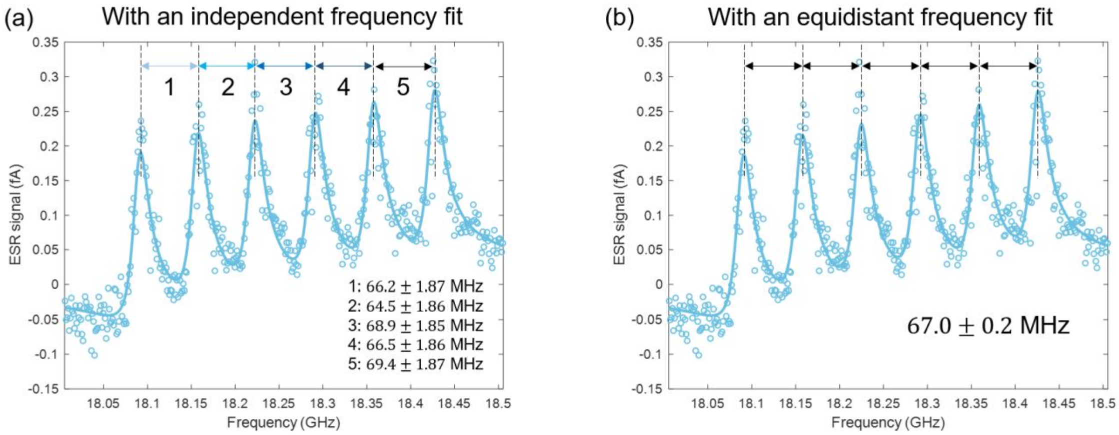

Figure S2. Hyperfine spectra (a) with an independent frequency fit and (b) with an equidistant frequency fit. In the fit function, the ESR frequency of each peak is given as a free parameter in (a), while the frequency splitting is fixed in (b). Error values are determined from 95% confidence interval of the fitting. We additionally consider the statistical error ~0.8 MHz as described in the main text. By independently fitting the frequencies of the ESR peaks in (a), we conclude that the distances between neighboring ESR peaks are identical within the error bars. The equidistant peaks indicate that nuclear Zeeman energy and the quadrupole interaction are negligible in our measurement (V_{DC} = 40 mV, I_{set} = 12 pA, V_{RF} = 20 mV, T = 0.6 K, and B_{ext} = 0.8 T).

Section 3. Hyperfine splitting as a function of external magnetic field direction measured with different atoms and tips

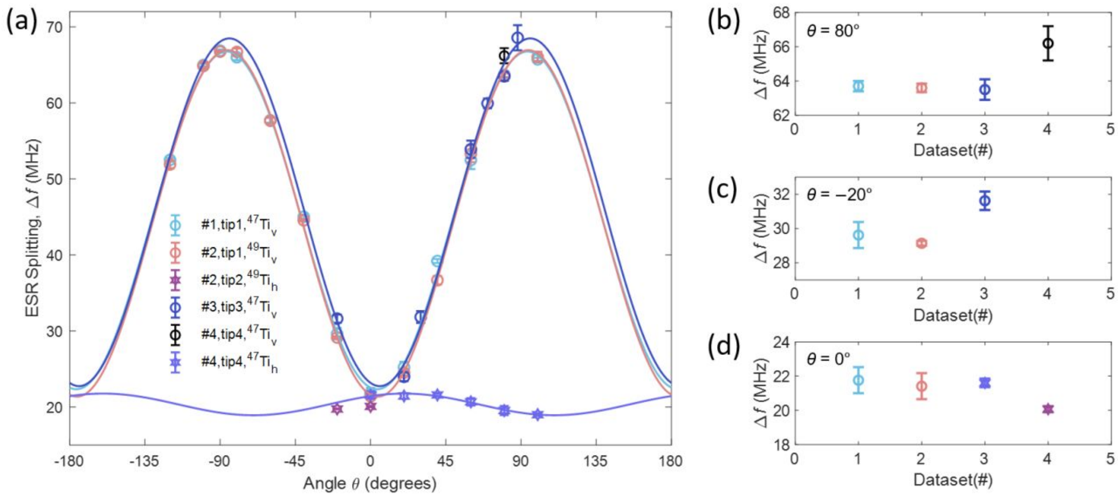

Figure S3. (a) Hyperfine splitting at different directions of external magnetic fields measured with different atoms and different tips. Four atoms labelled as #1–4 and four different tips labelled as tip1–tip4 were used in these measurements. Atoms on the same adsorption sites show similar hyperfine splitting regardless of the used tips. (b-d) Same data points as (a) but plotted at only certain angle of B_{ext} ($\theta = 80^\circ, -20^\circ, 0^\circ$). At the top panel, the dataset #4 was measured with at a higher current (I_{set} = 20 pA), which increases the broadening of the ESR peak, and lowers the accuracy of the extracted hyperfine splitting (see Sec. S5) (V_{DC} = 40 mV, I_{set} = 1.5~8 pA, V_{RF} = 15~50 mV, T = 0.6 K, and B_{ext} = 0.8 T).

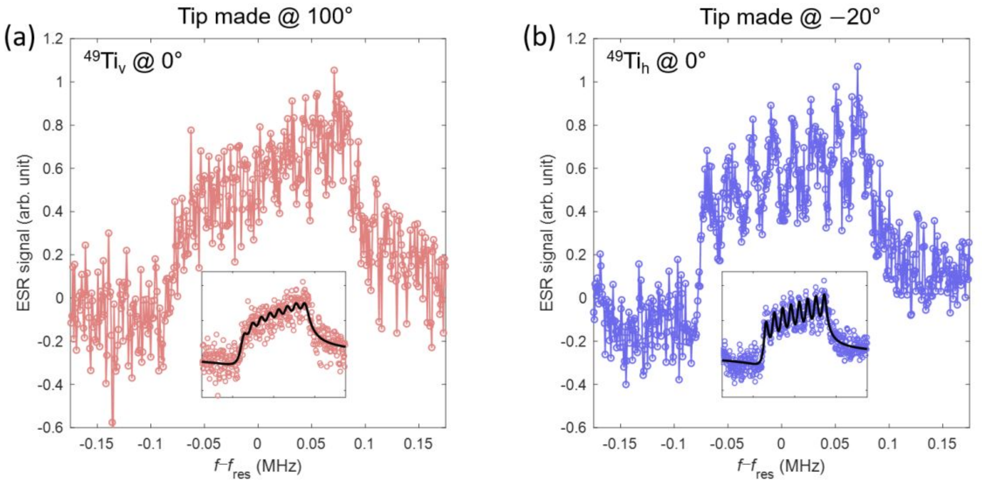

Figure S4. Hyperfine spectra of ^{49}Ti measured with an out-of-plane magnetic field ($\theta = 0^\circ$) for two different tips. The tips were prepared at different B_{ext} angles of (a) $\theta = 100^\circ$ (near in-plane direction) and (b) –20° (near out-of-plane direction). According to our previous work,[1] the ESR signal is largest when the magnetic field angle θ during ESR measurement is close to the condition at which the tip was prepared, while the signal intensity significantly decreases as the measurement angle changes until about 90° off from the tip preparation angle. In (a), the tip was prepared at $\theta = 100^\circ$ while the curve was measured at $\theta = 0^\circ$, which results in a poor resolution. In (b), the tip was prepared at $\theta = -20^\circ$, which provides well-resolved ESR signals when measured at $\theta = 0^\circ$. Thus, to resolve ESR splitting more clearly when the magnetic field is applied along the out-of-plane direction ($\theta = 0^\circ$), a spin-polarized tip prepared with a field applied close to $\theta = 0^\circ$ gives the best results. Insets: the fitted spectra reveal a more accurate splitting for the tip used in (b) (ESR condition: V_{DC} = 40 mV, I_{set} = 3 pA, V_{RF} = 30 mV, and T = 0.6 K).

Section 4. Hyperfine splitting as a function of the magnitude of the external magnetic field

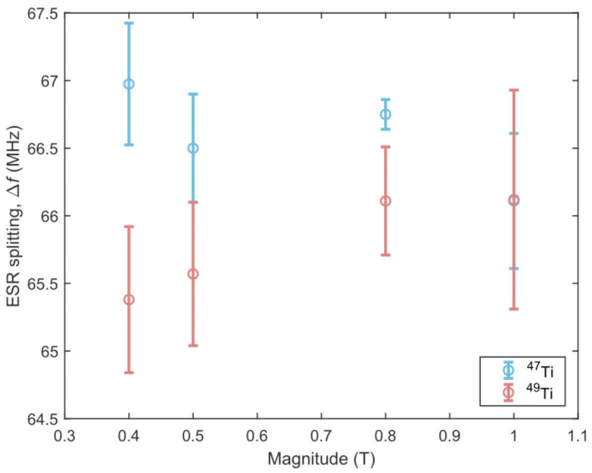

Figure S5. Hyperfine splittings of $^{47}Ti_v$ and $^{49}Ti_v$ measured at different external magnetic field intensities while keeping the field direction at $\theta = -90^\circ$.

In order to evaluate the contribution of the nuclear Zeeman interaction, we measured ESR spectra at different magnetic field magnitudes. As shown in Figure S5, no clear trend is observed from the data acquired on the two Ti isotopes. To rationalize this observation, we consider the spin Hamiltonian including the nuclear Zeeman interaction:

$$H = H_{EZ} + H_{HF} + H_{NZ} = \mu_B \boldsymbol{B}_{\text{ext}} \cdot g \cdot \boldsymbol{S} + \boldsymbol{S} \cdot \boldsymbol{A} \cdot \boldsymbol{I} + \mu_N \boldsymbol{B}_{\text{ext}} \cdot g_I \cdot \boldsymbol{I}, \quad \text{(S1)}$$

where H_{EZ}, H_{HF} and H_{NZ} are the electron Zeeman interaction, the hyperfine interaction, and the nuclear Zeeman interaction, respectively, μ_N is the nuclear magneton, and g_I is the g-factor of Ti nuclear spin.

To evaluate the magnitude of the nuclear Zeeman term, note that the nuclear magneton divided by the Planck constant is $\frac{\mu_N}{h} =$ 7.6226 MHz/T,[2] and the nuclear g-factors are g_I = –0.3154 and –0.3155 for ^{47}Ti and ^{49}Ti, respectively.[3] Therefore the nuclear gyrometric ratios are $\gamma_N =$ 2.404 MHz/T and 2.405 MHz/T for ^{47}Ti and ^{49}Ti, respectively, according to $\gamma_N = \frac{\mu_N \cdot g_I}{h}$. As a result, for both isotopes, at B_{ext} = 0.4 T, the nuclear Zeeman interaction is around 0.96 MHz, and at 1.0 T, the nuclear Zeeman interaction is around 2.4 MHz. This small energy shift is comparable to the error bars in our measurements (Figure S5) and significantly less than the total hyperfine interaction. We thus conclude that the nuclear Zeeman interaction can be neglected under the experimental conditions used in the main text (B_{ext} = 0.8 T).

Section 5. Hyperfine splitting as a function of tunnel current

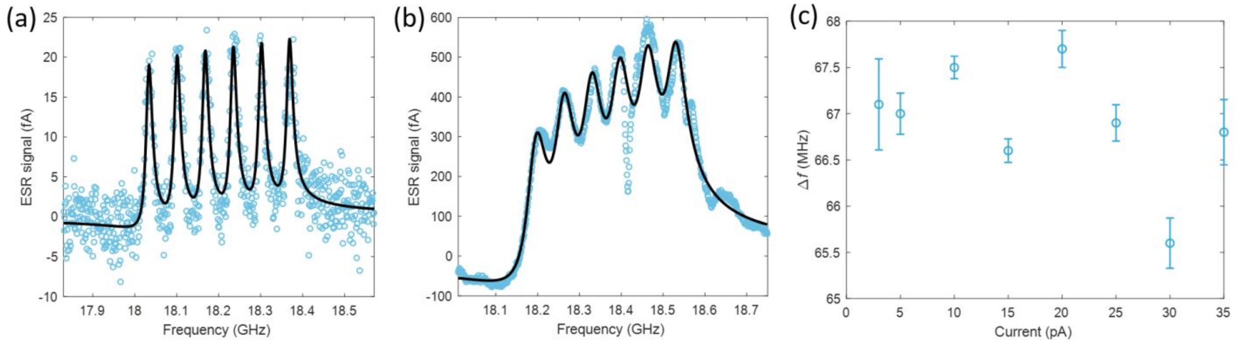

Figure S6. (a) ESR spectrum measured at I_{set} = 5 pA and (b) 30 pA (V_{DC} = 40 mV, V_{RF} = 30 mV and T = 0.6 K). (c) Hyperfine splitting of $^{47}Ti_v$ as a function of tunnel current extracted from measurements similar to (a) and (b), showing no clear trend. At lower tunnel current (below 8 pA), the ESR peaks are sharp and easily resolvable. At higher tunnel current (30 pA), significant peak broadening occurs due to decoherence by tunneling electrons[4] and leads to poorer fitting. Therefore, in the main text we use ESR spectra measured at a low tunnel current for the data analysis. Note that in this experiment, changing the tunnel current is obtained by changing the tip-sample distance. Although varying the tip-sample distance modifies the magnitude of tip's magnetic field, the variation of the magnetic field intensity has no significant impact on the total hyperfine splitting, as also discussed in Section 4.

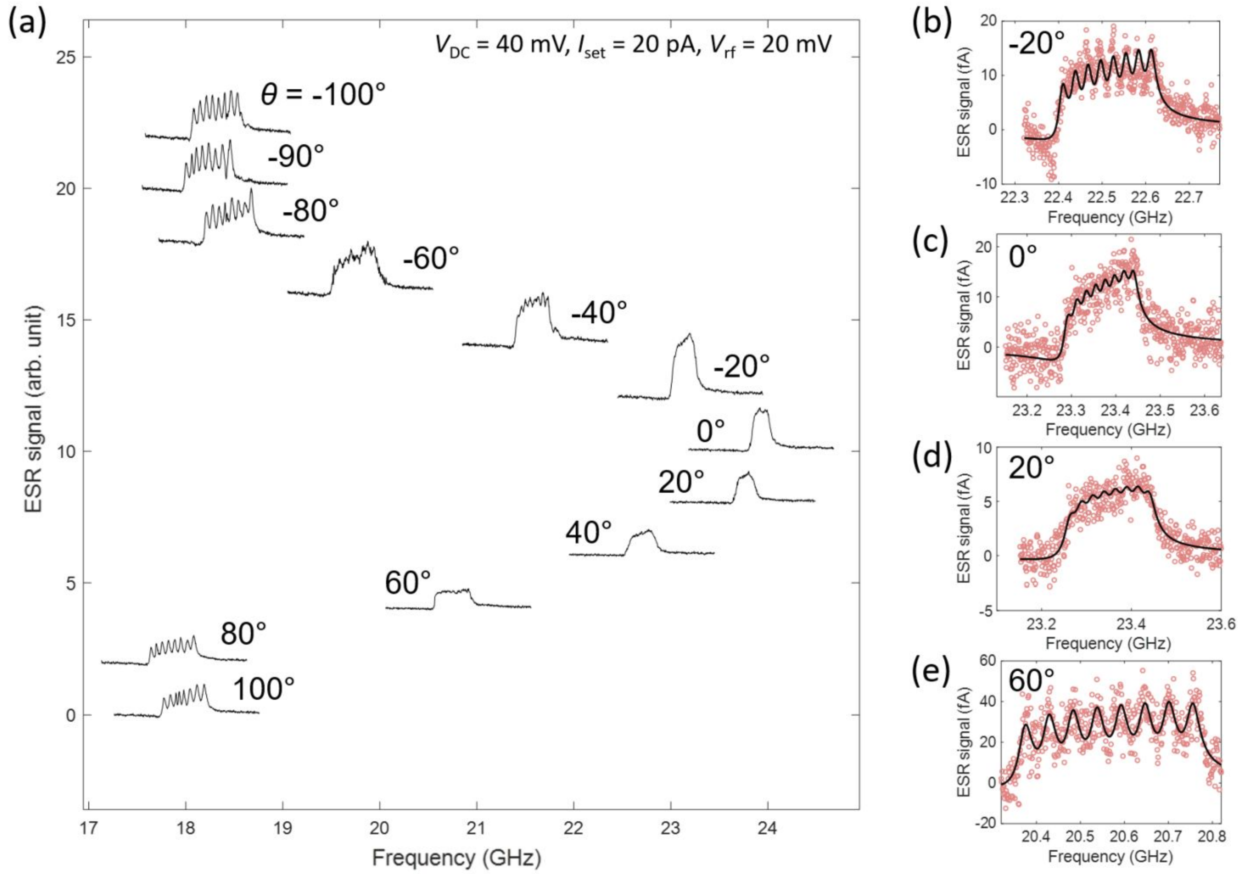

Figure S7. (a) ESR spectra measured on the same $^{49}Ti_v$ with the same tip at different angles of the external magnetic field. All spectra were measured under the same conditions ($V_{DC} = 40$ mV, $I_{set} = 20$ pA, and $V_{RF} = 20$ mV). Frequency shifts of the ESR resonance are due to g-factor anisotropy. The changes in the splittings are due to hyperfine anisotropy. (b–e) Higher-resolution ESR spectra at selected magnetic field angles (setpoint: $V_{DC} = 40$ mV, $I_{set} = 1.5\sim 8$ pA, $V_{RF} = 15\sim 50$ mV and $T = 0.6$ K).

Section 6. Density functional theory and EasySpin calculations of Ti isotopes on MgO on Ag(100)

The DFT calculations are performed using plane-wave basis as implemented in Quantum Espresso (V7.0).[5,6] We use PBE pseudopotentials from the PSLibrary[7] for all atomic types in our system and expand the basis with a cutoff of 70 Ry for the kinetic energy and 700 Ry for the charge density. Integration of the Brillouin zone is performed on a 4×4×1 k-grid with cold smearing of ~150 K.[8] All calculations use spin-polarization. We apply a Hubbard-U (U=2 eV) correction to the $3d$ states of Ti and account for dispersive forces using Grimme's D3 van der Waals correction.

Our structure consists of 4 monolayers (ML) of Ag (at equilibrium lattice constant $a_{\mathrm{Ag}}^{\mathrm{PBE}} = 4.15$ Å) covered by 2 ML of MgO expanded in a 3×3 lateral unit cell capped by 12 Å of vacuum in the out-of-plane $\hat{z}$-direction. The hydrogenated Ti is then positioned on a bridge site of MgO and the whole system is relaxed until all forces are $<10^{-4}$ Ry/a_0 (a_0 is the Bohr radius). GIPAW calculations use nuclear g-factors from the IAEA reference tables[9] of $g_N = -0.315\ \mu/I$ for ^{47}Ti and ^{49}Ti. The near identical nuclear g-factors are also consistent with the experimental results.

A hyperfine tensor is used to simulate spectra along different directions using EasySpin "pepper".[10,11] We assume the high-field limit for all our calculations, with the electron Zeeman energy as the dominant energy scale. No quadrupole contribution to the hyperfine splitting was discernible in the experiment for the hydrogenated Ti at a bridge-site, and hence quadrupolar moments were set to 0 in the simulations.

We note that the GIPAW hyperfine tensor obtained from DFT qualitatively agrees with the experiment as discussed in the main text but quantitatively deviates from the experiment. This is not surprising considering the dependence of the hyperfine tensor on the exchange-correlation functional and other factors.[12] We find that a simple angle-independent rescaling of the GIPAW results yields quantitatively agreement with the experimental results. A reasonable agreement can be reached by re-scaling the isotropic hyperfine interaction A_{iso} from 170.4 MHz to 35 MHz as shown in Figure S8a, S8d, and Table S1. The results can be further improved by separately re-scaling isotropic and anisotropic hyperfine interactions, which is performed by minimizing the deviation of the angle-dependent hyperfine splitting obtained in the experiment. As shown in Figure S8b and S8e, this procedure results in an overestimation of the coupling along $\hat{z}$ but nevertheless captures the important aspects of the anisotropy. Finally, in Figure S8c and S8f, we performed an optimization of the full hyperfine tensor by using the experimentally determined values. All values of the hyperfine tensors from DFT are listed in Table S1.

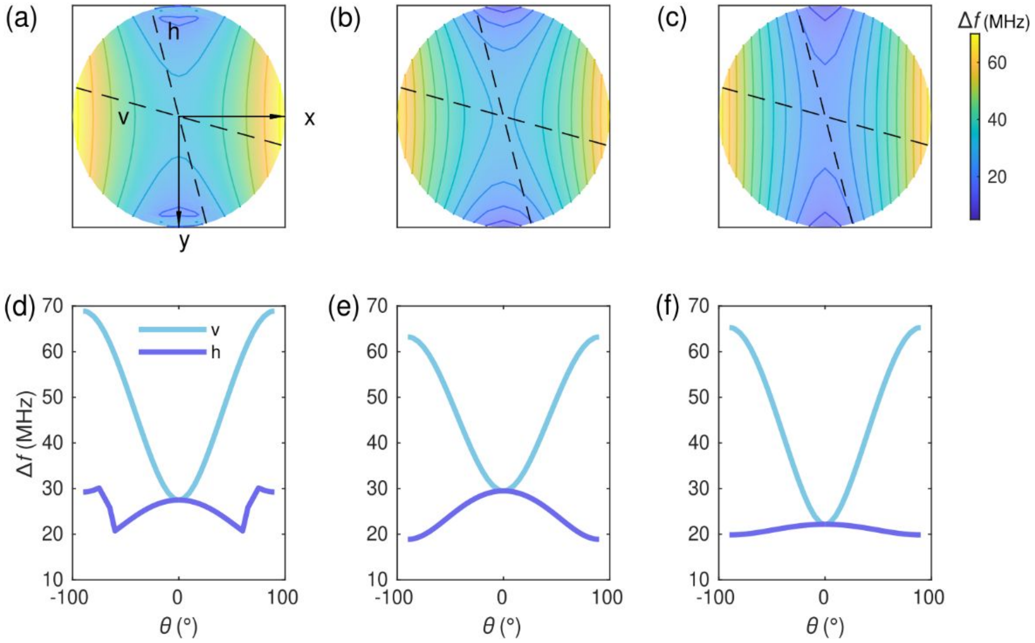

Figure S8. Simulated splitting of the hyperfine coupling along different (*xyz*) directions obtained for three different hyperfine tensors: (a) values obtained from DFT after rescaling only the isotropic hyperfine interaction (GIPAW-1), (b) values after rescaling both isotropic and anisotropic parts (GIPAW-2), and (c) values after an optimization of the full hyperfine tensor (GIPAW-3). *x*- and *y*-directions correspond to the O-O, Mg-Mg bonding directions, respectively, and the *z*-direction to the out-of-plane direction. (d–f) Simulated hyperfine splitting for comparison with main Figure 3f. v and h label the directions for vertical and horizontal Ti atoms, respectively. The directions used in (d)-(f) are indicated by dashed lines in (a)-(c). The sudden jump in (d) is related to the difficulty in extracting an exact value of the splitting when ESR spectra become malformed along these directions.

Table S1. Values for the hyperfine tensor (in MHz) and number of optimized parameters n_{opt}. GIPAW-0 corresponds to the values as obtained from DFT directly, GIPAW-1 is obtained by only rescaling n_{opt}=1 parameter (A_{iso}), GIPAW-2 by optimizing the DFT results by separately rescaling the isotropic and anisotropic parts, GIPAW-3 by directly optimizing the full hyperfine tensor starting from the bare DFT results.

Method	n_{opt}	A_{iso}	T_O	T_{Mg}	T_z
GIPAW-0	0	170.4	36.4	-30.2	-6.1
GIPAW-1	1	35.6	36.4	-30.2	-6.1
GIPAW-2	2	32.4	32.3	-26.8	-5.4
GIPAW-3	3	34.2	33.9	-21.8	-12.0

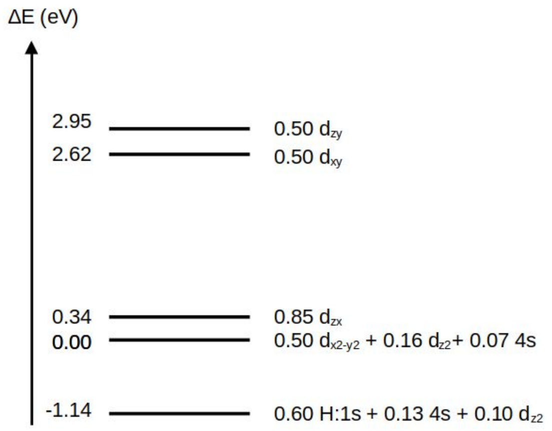

Figure S9. Schematic of the low-lying Ti states as obtained from DFT. The states are ordered in energy relative to the lowest state with significant Ti:3d weight. The state which is dominantly H:1s is shown for reference at around -1 eV. The labels on the right describe the dominant contributions from Ti atomic wave functions. The missing contributions are mostly spurious Ag, Mg and O contributions. We note that the exact composition of each state as well as its energy will depend on the particular function employed as well as corrections such as Hubbard U.

Reference

1. Kim, J.; Jang, W.-j.; Bui, T. H.; Choi, D.-J.; Wolf, C.; Delgado, F.; Chen, Y.; Krylov, D.; Lee, S.; Yoon, S.; Lutz, C. P.; Heinrich, A. J.; Bae, Y., Spin resonance amplitude and frequency of a single atom on a surface in a vector magnetic field. *Phys. Rev. B* **2021,** 104, (17), 174408.

2. Tiesinga, E.; Mohr, P. J.; Newell, D. B.; Taylor, B. N., CODATA recommended values of the fundamental physical constants: 2018. *Journal of Physical and Chemical Reference Data* **2021,** 50, (3), 033105.

3. W. M. Haynes, D. R. L., Thomas J. Bruno, *CRC Handbook of Chemistry and Physics*. 97th Edition ed.; CRC Press: Boca Raton, 2016; p 2670.

4. Willke, P.; Paul, W.; Natterer, F. D.; Yang, K.; Bae, Y.; Choi, T.; Fernández-Rossier, J.; Heinrich, A. J.; Lutz, C. P., Probing quantum coherence in single-atom electron spin resonance. *Sci. Adv.* **2018,** 4, (2), eaaq1543.

5. Giannozzi, P.; Andreussi, O.; Brumme, T.; Bunau, O.; Buongiorno Nardelli, M.; Calandra, M.; Car, R.; Cavazzoni, C.; Ceresoli, D.; Cococcioni, M.; Colonna, N.; Carnimeo, I.; Dal Corso, A.; de Gironcoli, S.; Delugas, P.; DiStasio, R. A.; Ferretti, A.; Floris, A.; Fratesi, G.; Fugallo, G.; Gebauer, R.; Gerstmann, U.; Giustino, F.; Gorni, T.; Jia, J.; Kawamura, M.; Ko, H. Y.; Kokalj, A.; Küçükbenli, E.; Lazzeri, M.; Marsili, M.; Marzari, N.; Mauri, F.; Nguyen, N. L.; Nguyen, H. V.; Otero-de-la-Roza, A.; Paulatto, L.; Poncé, S.; Rocca, D.; Sabatini, R.; Santra, B.; Schlipf, M.; Seitsonen, A. P.; Smogunov, A.; Timrov, I.; Thonhauser, T.; Umari, P.; Vast, N.; Wu, X.; Baroni, S., Advanced capabilities for materials modelling with Quantum ESPRESSO. *J. Phys.: Condens. Matter* **2017,** 29, (46), 465901.

6. Giannozzi, P.; Baroni, S.; Bonini, N.; Calandra, M.; Car, R.; Cavazzoni, C.; Ceresoli, D.; Chiarotti, G. L.; Cococcioni, M.; Dabo, I.; Dal Corso, A.; de Gironcoli, S.; Fabris, S.; Fratesi, G.; Gebauer, R.; Gerstmann, U.; Gougoussis, C.; Kokalj, A.; Lazzeri, M.; Martin-Samos, L.; Marzari, N.; Mauri, F.; Mazzarello, R.; Paolini, S.; Pasquarello, A.; Paulatto, L.; Sbraccia, C.; Scandolo, S.; Sclauzero, G.;

Seitsonen, A. P.; Smogunov, A.; Umari, P.; Wentzcovitch, R. M., QUANTUM ESPRESSO: a modular and open-source software project for quantum simulations of materials. *J. Phys.: Condens. Matter* **2009,** 21, (39), 395502.

7. Dal Corso, A., Pseudopotentials periodic table: From H to Pu. *Computational Materials Science* **2014,** 95, 337-350.

8. Marzari, N.; Vanderbilt, D.; De Vita, A.; Payne, M. C., Thermal Contraction and Disordering of the Al(110) Surface. *Phys. Rev. Lett.* **1999,** 82, (16), 3296-3299.

9. Stone, N. J., Table of nuclear magnetic dipole and electric quadrupole moments. *At. Data Nucl. Data Tables* **2005,** 90, (1), 75-176.

10. Stoll, S.; Schweiger, A., EasySpin, a comprehensive software package for spectral simulation and analysis in EPR. *Journal of Magnetic Resonance* **2006,** 178, (1), 42-55.

11. Stoll, S., *Electron Paramagnetic Resonance Investigations of Biological Systems by Using Spin Labels, Spin Probes, and Intrinsic Metal Ions, Part A*, Academic Press: Cambridge, Massachusetts, **2015**, Vol. 563, pp 121-142.

12. Tosoni, S.; Pacchioni, G., Magnetic nature and hyperfine interactions of transition metal atoms adsorbed on ultrathin insulating films: a challenge for DFT. *Physical Chemistry Chemical Physics* **2022,** 24, (26), 15891-15903.
